# Sensitivity of Arterial Spin Labeling for Characterization of Longitudinal Perfusion Changes in Frontotemporal Dementia and Related Disorders

**DOI:** 10.1016/j.nicl.2021.102853

**Published:** 2021-10-07

**Authors:** Tracy Ssali, Udunna C. Anazodo, Lucas Narciso, Linshan Liu, Sarah Jesso, Lauryn Richardson, Matthias Günther, Simon Konstandin, Klaus Eickel, Frank Prato, Elizabeth Finger, Keith St. Lawrence

**Affiliations:** a Lawson Health Research Institute, London, Canada; b Department of Medical Biophysics, Western University, London, Canada; c St. Joseph’s Health Care, London, Canada; d Fraunhofer Institute for Medical Image Computing MEVIS, Bremen, Germany; e University Bremen, Bremen, Germany; f Mediri GmbH, Heidelberg, Germany; g Department of Clinical Neurological Sciences, Western University, London, Canada

**Keywords:** Frontotemporal Dementia (FTD), Cerebral Blood Flow (CBF), Arterial Spin Labeling (ASL), Arterial Transit Time (ATT), Reproducibility

## Abstract

**Background:**

Advances in the understanding of the pathophysiology of frontotemporal dementia (FTD) and related disorders, along with the development of novel candidate disease modifying treatments, have stimulated the need for tools to assess the efficacy of new therapies. While perfusion imaging by arterial spin labeling (ASL) is an attractive approach for longitudinal imaging biomarkers of neurodegeneration, sources of variability between sessions including arterial transit times (ATT) and fluctuations in resting perfusion can reduce its sensitivity. Establishing the magnitude of perfusion changes that can be reliably detected is necessary to delineate longitudinal perfusion changes related to disease processes from the effects of these sources of error.

**Purpose:**

To assess the feasibility of ASL for longitudinal monitoring of patients with FTD by quantifying between-session variability of perfusion on a voxel-by-voxel basis.

**Methods and materials:**

ASL data were collected in 13 healthy controls and 8 patients with FTD or progressive supra-nuclear palsy. Variability in cerebral blood flow (CBF) by single delay pseudo-continuous ASL (SD-pCASL) acquired one month apart were quantified by the coefficient of variation (CV) and intraclass correlation coefficient (ICC). Additionally, CBF by SD-pCASL and ATT by low-resolution multiple inversion time ASL (LowRes-pCASL) were compared to Hadamard encoded sequences which are able to simultaneously measure CBF and ATT with improved time-efficiency.

**Results:**

Agreement of grey-matter perfusion between sessions was found for both patients and controls (CV = 10.8% and 8.3% respectively) with good reliability for both groups (ICC > 0.6). Intensity normalization to remove day-to-day fluctuations in resting perfusion reduced the CV by 28%. Less than 5% of voxels had ATTs above the chosen post labelling delay (2 s), indicating that the ATT was not a significant source of error. Hadamard-encoded perfusion imaging yielded systematically higher CBF compared to SD-pCASL, but produced similar transit-time measurements. Power analysis revealed that SD-pCASL has the sensitivity to detect longitudinal changes as low as 10% with as few as 10 patient participants.

**Conclusion:**

With the appropriate labeling parameters, SD-pCASL is a promising approach for assessing longitudinal changes in CBF associated with FTD.

## Highlights

• This study demonstrates the value of ASL for longitudinal monitoring of perfusion in FTD patients.
• Good agreement was found in repeat measures of CBF in patients and controls.
• Transit times were not a significant source of error for the selected post labeling delay (2 s).

## Nomenclature

**Acronyms**
3D: 3-Dimensional
3 T: 3 Tesla
ABS: Absolute Perfusion
aCBF: Absolute Perfusion
ACE: Addenbrooke's Cognitive Examination
AD: Alzheimer's Dieses
ANOVA: Analysis of Variance
ASL: Arterial Spin Labeling
ATT: Arterial Transit Time
BS: Between-Session
BSub: Between-Subject
CBF: Cerebral Blood Flow
CBI: Cognitive Behavioural Intervention
conv_TE-pCASL: Conventional Time-Encoded Pseudo Continuous Arterial Spin Labeling
CV: Coefficient of Variation
ENABLE: ENhancement of Automated Blood fLow Estimates
FBI: Frontal Behavioral Inventory
FDG: Fluorodeoxyglucose
FL_TE-pCASL: Free-Lunch Time-Encoded Pseudo Continuous Arterial Spin Labeling
FMRIB: Functional Magnetic Resonance Imaging of the Brain
FNIRT: FMRIB's nonlinear image registration tool
FOV: Field of View
FSL: FMRIB Software Library
FTD: Frontotemporal Dementia
FTLD: Frontotemporal lobar degeneration
GRASE: GRadient And Spin Echo
GRN: Granulin Gene
ICC: Intra-Class Correlation Coefficient
LD: Labelling Duration
LowRes-pCASL: Low-Resolution Pseudo Continuous Arterial Spin Labeling
M0: Equilibrium Magnetization
MAPT: Microtubule Associated Protein Tau
MATLAB: Matrix Laboratory
MCI: Mild Cognitive Impairment
MNI: Montreal Neurological Institute
MPRAGE: Magnetization Prepared - RApid Gradient Echo
MRI: Magnetic Resonance Imaging
NPI: Neuropsychiatric Inventory
pCASL: Pseudo Continuous Arterial Spin Labeling
PET: Positron Emission Tomography
PGRN: Progranulin
PLD: Post Labeling Delay
PPA: Primary Progressive Aphasia
PSP: Progressive Supra Nuclear Palsy
rCBF: Relative Perfusion
REL: Relative Perfusion
ROI: Region of Interest
SD: Standard Deviation
SD-pCASL: Single Delay Pseudo Continuous Arterial Spin Labeling
SIENA: Structural Image Evaluation, using Normalisation, of Atrophy
SNR: Signal to Noise Ratio
SPM: Statistical Parametric Mapping
T1: Longitudinal Relaxation Rate
T2: Transverse Relaxation Rate
TDP: Transactive response DNA-binding protein
TE: Echo Time
TI: Inversion Time
TR: Repetition Time
WS: Within-Session

## 1 Introduction

Frontotemporal dementia (FTD) and related disorders comprise a clinically and pathologically heterogeneous group of neurodegenerative disorders that are characterized by progressive atrophy of the frontal and temporal lobes with relative sparing of the posterior cerebral regions, and abnormal molecular accumulations, mostly commonly of tau or TDP-43 (Seelaar et al., 2011). FTD is the second most common form of early onset dementia, with the greatest prevalence among individuals between 45 and 64 years of age (Finger, 2016). FTD is highly heritable, with up to 40 percent of cases considered hereditary, and ∼ 15% autosomal dominantly inherited (Warren et al., 2013). Advances in the understanding of the link between genetic factors and the underlying pathophysiology (Whitwell and Josephs, 2012, Whitwell et al., 2009), and subsequently, the development of candidate disease modifying treatments, have stimulated the need for efficient tools to assess treatment efficacy (Logroscino et al., 2019)**.**

Longitudinal neuroimaging studies can provide insight into therapeutic efficacy and characterize natural disease trajectory, which could increase the possibility of presymptomatic intervention. The majority of studies assessing longitudinal brain function in FTD have focused on assessing tissue volume loss by structural magnetic resonance imaging (MRI) (Whitwell et al., 2015, Chan et al., 2001, Jiskoot et al., 2019) or regional glucose hypometabolism by ^18^F fluorodeoxyglucose (FDG) positron emission tomography (PET) (Schuster et al., 2015, Rajagopalan and Pioro, 2019, Diehl-Schmid et al., 2007). While these approaches are used diagnostically, for longitudinal imaging, structural changes are subtle at the early disease stage and PET imaging is expensive and access limited (Piguet et al., 2011, Olm et al., 2016, Verfaillie et al., 2015).

Due to the coupling of perfusion and metabolism, an attractive alternative to FDG PET is the MRI-based perfusion imaging technique, arterial spin labeling (ASL) (Verfaillie et al., 2015, Tosun et al., 2016). In FTD, perfusion changes precede structural findings, with reduced frontal and temporal cerebral blood flow (CBF) indexed by ASL in presymptomatic FTD mutation carriers (Dopper et al., 2016, Mutsaerts et al., 2019) as well as symptomatic FTD (Olm et al., 2016). Furthermore, longitudinal reductions in perfusion by ASL have been detected in FTD patients (Staffaroni et al., 2019a) and are associated with clinical measures of cognitive decline (Staffaroni et al., 2019b), highlighting the potential for ASL for longitudinal assessments. However, despite that perfusion is one of the earliest changes in pathological aging (Mutsaerts et al., 2019, Iturria-Medina et al., 2016), beyond the aforementioned studies, there are few reports in the literature investigating the stability of ASL for longitudinal imaging of FTD patients and no studies assessing the sensitivity of ASL for detecting longitudinal perfusion changes in FTD patients.

In comparison to young healthy populations where ASL shows good reliability and reproducibility (Chen et al., 2011, Parkes et al., 2004, Murphy et al., 2011), perfusion measurements among elderly populations can be challenging due to reduced signal to noise ratio (SNR) and potential vascular changes (Kilroy et al., 2014, Xu et al., 2010). With age, the brain’s major feeding vessels become tortuous and the prevalence of stenosis increases. These factors can result in underestimated CBF due to increases in the arterial transit time (ATT) – i.e., the time required for labeled water to travel from the labeling site to brain tissue – greatly reducing the sensitivity of ASL to detect clinically relevant perfusion changes (Dai et al., 2017). Furthermore, by collecting data on different days, sources of variation attributed to repositioning and differences in resting perfusion between days can introduce error that can confound measurement of longitudinal change. Establishing the magnitude of perfusion changes that can be reasonably detected among older control and patient populations would help address the influence of sources of between scan variability (Clement et al., 2018), namely, transit time and repositioning errors, as well as day-to-day fluctuations in CBF.

The primary aim of this study was to assess the sensitivity of ASL for detecting longitudinal changes in perfusion among patients with FTD using optimized parameters based on the ASL white paper (Alsop et al., 2015). To assess sources of variability, reproducibility and reliability of single delay pseudo continuous ASL (SD-pCASL) were assessed for same-day scans and scans collected during sessions separated by a month. A relatively short between-session period (4 weeks) was selected to avoid disease-related brain atrophy or pathological changes in the patient population. Differences in variability between the aforementioned scan separations reflects error due to repositioning and differences in resting perfusion between sessions. To assess the effects of day-to-day changes in perfusion, variability was assessed for absolute and relative perfusion. Power analysis was conducted to determine the number of participants required to detect clinically relevant longitudinal perfusion changes. The influence of ATT on the longitudinal reproducibility of CBF, was determined by quantifying the between-session variability of ATT measured by a low-resolution (LowRes-pCASL) sequence using multiple inversion times. Given that visual assessment remains a primary source of scan interpretation, a voxel-by-voxel approach was implemented to visualize the spatial distribution of variability and, furthermore, to identify regions where longitudinal changes would be more challenging to detect. As previous studies have suggested that time-encoded multi-delay sequences can improve SNR and temporal efficiency (Samson-himmelstjerna et al., 2016, Guo et al., 2018, Dai et al., 2013), a secondary aim was to compare perfusion and transit times measured by SD-pCASL and LowRes-pCASL, respectively, to Hadamard-encoded multi-delay sequences.

## 2 Methods

### 2.1 Participants

This study was approved by the Western University Health Sciences Research Ethics Board and was conducted in accordance with the Declaration of Helsinki ethical standards. Participants provided written informed consent in compliance with the Tri-Council Policy Statement of Ethical Conduct for Research Involving Humans.

Fourteen neurologically healthy controls and ten patients with FTD or progressive supra-nuclear palsy (PSP) were enrolled in the study. Patients were recruited through the Cognitive Neurology and Aging Brain Clinic at Parkwood Hospital (St Joseph’s Health Care London) and controls were recruited through advertisements and the clinic’s volunteer pool. Studies were performed between November 2019 and December 2020. The patient cohort consisted of individuals meeting the consensus criteria for probable or definite FTD or PSP; specifically, behavioural variant (bvFTD) (Rascovsky et al., 2011), semantic variant (svFTD) (Gorno-Tempini et al., 2011), nonfluent primary progressive aphasia (nfPPA) (Gorno-Tempini et al., 2011), and PSP (Höglinger, 2017). Exclusion criteria included (1) any significant neurologic disease other than suspected FTD, (2) presence of pacemakers, aneurism clip, artificial heart valves, ear implants, metal fragments or foreign objects that would preclude MRI participation, (3) major depression, bipolar disorder, psychotic features or behavioural problems, and (4) any significant systemic illness or unstable medical condition. Diagnostic evaluations were performed by a clinical neurologist (E.F) based on clinical evaluation, neurocognitive testing, clinical MRI brain imaging, and genetic testing.

### 2.2 Measures

Patients completed a battery of validated cognitive tasks assessing domains of cognition including the Addenbrooke's Cognitive Examination (ACE-III American Version A) (NeurRA; www.neura.edu.au) (Mioshi et al., 2006), Mini-ACE (M-ACE; NeurRA), Boston Naming Test Second Edition Short Form (BNT) (Kaplan et al., 1983), Short Form Geriatric Depression Scale (GDS) (Sheikh and Yesavage, 1986). Study partners completed ratings of participants’ symptoms and behaviour using the Cambridge Behavioural Inventory (CBI) (Wedderburn et al., 2008), Neuropsychiatric Inventory (NPI) (Cummings et al., 1994), Frontal Behavioural Inventory (FBI) (Kertesz et al., 2000), and Cornell Scale for Depression (Alexopoulos et al., 1988) during one of their two visits.

### 2.3 Imaging

All MRI examinations were performed on a 3 T Siemens Biograph mMR scanner using a 12-channel head coil. Participants were required to abstain from caffeine 8 h before each scan. Each participant was scanned on two occasions separated by approximately 4 weeks**.** Repeat scans were scheduled at a similar time of day to minimize time-of-day effects (Parkes et al., 2004). SD-pCASL data were acquired twice during each imaging session for a total of 4 scans. This protocol allowed for the assessment of two types of within-subject variability: within-session, representing fluctuations in same-day measurements, and between-session, representing the variability in measurements separated by 4 weeks. LowRes-pCASL was performed once in each session to assess between-session variability in transit times. Hadamard-encoded sequences were acquired once during one of the two imaging sessions. All scans were performed at rest with the participants awake in the scanner. To improve compliance, participants watched a low cognitive demand movie (Vanderwal et al., 2015). Each scanning session included a T1-weighted magnetization-prepared rapid acquisition gradient echo (MPRAGE) sequence with repetition time (TR)/echo time (TE): 2000/2.98 ms, voxel size: 1 mm isotropic, field of view (FOV) 256 × 256 × 176 mm^3^, scan time: 4:38 min.

#### 2.3.1 Single delay pCASL

Single delay pCASL data were acquired with a 4-shot 3D gradient and spin echo (GRASE) readout (Günther et al., 2005); TR/TE: 4500/22.14 ms, voxel-size: 4 mm isotropic, FOV: 256 × 256 × 128 mm^3^, label-control pairs: 8, bandwidth: 2298 Hz/Px, 1 preparing scan, scan time: 4:53 min. A post-labeling delay (PLD) of 2000 ms and label duration (LD) of 1800 ms were used as recommended by the ASL consensus paper (Alsop et al., 2015). For all pCASL sequences, two inversion pulses were used to null components with relaxation times T1 = 700 and 1400 ms (Günther et al., 2005). To maintain a consistent acquisition protocol, these parameters were used in both patients and healthy controls, despite the shorter recommended PLD for healthy controls. An equilibrium magnetization image (M0) was acquired to convert perfusion-weighted images into physiological units of blood flow. Imaging parameters were identical to the pCASL acquisition except for a TR of 7000 ms and no background suppression or labeling. Since the feeding arteries are often tortuous in elderly populations (Dai et al., 2017, Farkas and Luiten, 2001), the labeling plane offset was manually adjusted for each participant to ensure the labeling plane was straight and parallel to the vessels. A 3D time-of-flight MRI angiography (TR/TE: 22.0/3.75 ms, voxel-size: 0.3 × 0.3 × 1.5 mm^3^, FOV: 263 × 350 × 350 mm^3^, 4 slabs, 30 slices per slab, scan time: 4:23 min) was acquired to identify the major arteries for labeling plane preparation. The offset ranged between 90 and 125 mm from the center of the imaging slab. The same labeling plane was used for all subsequent ASL sequences.

#### 2.3.2 Low-Resolution multi-delay pCASL

LowRes-pCASL data were acquired with a single-shot 3D GRASE readout: TR/TE: 5500/20.68 ms, voxel-size: 7.8 mm isotropic, FOV: 255 × 500 × 125 mm^3^, PLDs: 700, 1300, 1900, 2500, 3000 ms, LD: 2000 ms, bandwidth: 2298 Hz/Px, 1 preparing scan. Four label-controls pairs were acquired for each PLD for a total scan time of 4:10 min. These images were acquired to map the ATT.

#### 2.3.3 Hadamard encoded multi-delay pCASL

With Hadamard-encoded ASL, the pCASL bolus is divided into several uniquely encoded label and control sub-boluses where their ordering corresponds to a Hadamard matrix (Samson-himmelstjerna et al., 2016, Teeuwisse et al., 2014). Two variations of this sequence were employed: (1) conventional time-encoding (conv_TE-pCASL), where the sub-boluses are divided into blocks of equal length, and (2) free-lunch time-encoding (FL_TE-pCASL), where the labeling period is similar in length to the SD-pCASL sequence; however, the traditional PLD is replaced with time-encoding blocks. By linearly combining the images, perfusion-weighted images at different PLDs were extracted.

Hadamard-encoded data were acquired with a 2-shot 3D-GRASE readout sequence with 8 encoding steps, TR/TE: 5500/21.22 ms, voxel-size: 5 mm isotropic, FOV: 320 × 215 × 120 mm^3^, bandwidth: 2894 Hz/Px, slice partial Fourier: 6/8, phase partial Fourier: 6/8, 4 measurements per PLD. For conv_TE-pCASL, the sub-bolus duration was 400 ms and effective PLDs were 2600, 2200, 1800, 1400, 1000, 600, 200 ms. For FL_TE-pCASL, the sub-bolus duration was 250 ms, a free-lunch LD of 2000 ms, and PLD = 200 ms. Altogether, this corresponded to PLD_1_/LD_1_: 1700 ms/2000 ms, LD_2-7_: 250 ms, PLD_2-7_: 1450, 1200, 950, 700, 450, 200 ms. The total scan time for both sequences was 5:52 min. An M0 image was acquired with identical parameters except no background suppression or labeling.

### 2.4 Image processing

Image analysis was performed with SPM12 (http://www.fil.ion.ucl.ac.uk) (Ashburner, 2012), Oxford Centre for Functional MRI of the Brain (FMRIB)'s software library (FSL 6.0.1) (Jenkinson et al., 2012), and in-house MATLAB scripts ( The MathWorks, Natick, MA). Prior to any analysis, all images were manually reoriented to the axis of the anterior and posterior commissure. T1-weighted images from each session were coregistered and averaged using SIENA (Smith et al., 2002). By transforming the two structural images into a halfway space, both images undergo the same resampling steps, thereby reducing potential interpolation bias. The resulting structural images were processed using the fsl_anat pipeline to generate bias-corrected, skull-stripped, tissue-segmented, and spatially normalized structural images, as well as a normalization matrix (Smith, 2004). Grey and white matter masks were generated by thresholding the respective tissue segmented images to include voxels with tissue probabilities >0.8.

#### 2.4.1 Single delay pCASL

M0 images from the first and second imaging session were realigned to their mean and co-registered to the T1-weighted images. Using SPM12, raw SD-pCASL data were motion corrected, registered to the mean M0 and pairwise subtracted. Poor quality difference images were identified using ENABLE (Shirzadi et al., 2018), an automated sort/check algorithm. Briefly, each difference image was scored based on a linear combination of ASL quality features: temporal SNR, detectability metric (proportion of grey-matter voxels with signals significantly greater than zero), temporal contrast-to-noise ratio, and spatial coefficient of variation. Image volumes that did not meet the quality criterion were removed. Perfusion was quantified using the Oxford ASL toolbox (oxasl) which uses Bayesian inference to perform kinetic modeling and spatial regularization (Chappell et al., 2009, Groves et al., 2009). The incorporation of these spatial and biophysical priors reduces the uncertainty of model parameters by encoding realistic assumptions and accounting for natural variability in the model parameters. A standard well-mixed single compartment model was applied to the motion-corrected and filtered perfusion-weighted images (Buxton, 2005). Model parameters were based on the guidelines of the ASL consensus paper (Alsop et al., 2015): T_1_ of tissue = 1300 ms (Wansapura et al., 1999), T_1_ of arterial blood = 1650 ms (Zhang et al., 2013), labeling efficiency = 0.85 (Dai et al., 2008), blood–brain partition coefficient = 0.9 ml/g (Herscovitch and Raichle, 1985). Registration between perfusion/transit time and structural images was carried out using boundary-based registration (Jenkinson et al., 2002). Images were normalized to the MNI template by applying the transformation parameters generated by fsl_anat using a non-linear image registration tool (FNIRT Jenkinson and Smith, 2001) and smoothed by a 6-mm Gaussian filter.

#### 2.4.2 Multi-Delay pCASL

Raw LowRes-pCASL data were motion corrected and pairwise subtracted using SPM12. Data were fit to the general kinetic model with oxasl to extract the ATT (Chappell et al., 2009). The single compartment model with no dispersion was fit using the aforementioned SD-pCASL model parameters. ENABLE was implemented to remove low quality difference images. Hadamard-encoded data were processed in a similar manner except no motion correction was applied and instead of pairwise subtraction, the Hadamard transform with Walsh ordering was applied to generate images for each label/sub-bolus (Samson-himmelstjerna et al., 2016). Both perfusion and ATT maps were generated from the Hadamard sequences. All resulting data were normalized to the MNI template and smoothed as described previously.

### 2.5 ROI analysis

Region of interest (ROI) analysis was performed to assess regional reproducibility of SD-pCASL and compare CBF and ATT measured by the different sequences. This was performed in grey and white matter as well as regions commonly associated with FTD; namely, the orbitofrontal gyrus, inferior frontal gyrus, superior frontal gyrus, insular cortex, amygdala, temporal pole, and occipital gyrus (as a reference region) (Anazodo et al., 2018). FTD-specific ROIs were generated by combining regions from the automated anatomical labeling (aal) atlas in WFU Pickatlas (Wake Forest University, http://fmri.wfubmc.edu/cms/software).

### 2.6 Statistics

Statistical analysis was performed using R (R Core Team 2013, https://www.r-project.org/) and MATLAB. Variance components were estimated using a random effects model that employed restricted maximum likelihood. The variance components were estimated according to the following model (Shavelson and Webb, 1991):

(1) $${\mathrm{CBF}}_{\mathrm{ijk}}=\mu +U_{i}+V_{\mathrm{ij}}+\varepsilon_{\mathrm{ijk}}$$

This model was fit with perfusion (CBF_ijk_) for the i^th^ subject, j^th^ session, and k^th^ run, as the response variable and random effects for subject (U) and subject-by-session (V). The grand mean is represented by μ and ε is the residual. By nesting session within subject, sessions were uniquely coded to each subject. Variance was decomposed into 3 components: variance between subjects, variance between sessions, and a residual variance component due to random error. This residual term is an estimate of variance that would result from the two repeat scans within a single session for a given participant. Each variance component indicates the magnitude of variance that the respective individual factor contributes. The within-subject variance (i.e., sum of the between-session and within-session variances) represents the variance in perfusion images acquired during sessions collected 4 weeks apart in a given participant. This estimate reflects the variance encountered in a study in which a participant is scanned once in each session.

Reproducibility, hereby defined as the variability in repeat measurements, was quantified by the coefficient of variation (CV). Between-subject and within-subject (i.e. within/between-session) CV were calculated by dividing the respective standard deviation (σ) by the mean (μ):

(2) $$\mathrm{CV}=\frac{\sigma }{\mu }*100\%$$

The intraclass correlation coefficient (ICC) was used to assess reliability (Shrout and Fleiss, 1979). While the ICC can be interpreted as the variance in the outcome variable that is accounted for by the grouping variable (e.g. subjects), an alternate interpretation is the expected correlation between randomly drawn units from the same group (Hox et al., 2018). In the context of the current study, we implement two variants of the ICC; ICC_between_, defined as the expected correlation in CBF among sessions for a randomly selected subject and ICC_within_, defined as the estimated correlation in CBF from runs within the same session for a randomly selected subject. These metrics of reliability were assessed by:

(3) $$\mathrm{IC}C_{\mathrm{between}}=\frac{\sigma_{\mathrm{subject}}^{2}}{\sigma_{\mathrm{total}}^{2}}$$

(4) $$\mathrm{IC}C_{\mathrm{within}}=\frac{\sigma_{\mathrm{subject}}^{2}+\sigma_{\mathrm{session}-by-subject}^{2}}{\sigma_{\mathrm{total}}^{2}}$$

Total variance was defined as the sum of the individual components:

(5) $$\sigma_{\mathrm{total}}^{2}=\sigma_{\mathrm{subject}}^{2}+\sigma_{\mathrm{session}-by-subject}^{2}+\sigma_{\mathrm{residual}}^{2}$$

ICC values range between 0 and 1, where results were interpreted based on the following guidelines (Cicchetti and Sparrow, 1981): poor (<0.4), fair (0.41–0.59), good (0.6 – 0.74), and excellent (>0.75). Reproducibility and reliability were calculated on a voxel-by-voxel basis to visualize the spatial distribution.

Based on the variance in CBF derived by the random effects model, power calculations were performed to estimate the number of participants (N) required to detect a given change (Δ) in perfusion between sessions. This was computed by the following equation:

(6) $$N=\frac{2\sigma^{2}{\left(Z_{1-\alpha }+Z_{1-\beta }\right)}^{2}}{\Delta^{2}}$$

where σ^2^represents the within-subject variance, Z_1-α_ is the Z-score for the significance criterion, and Z_1- β_ is the z-score for the statistical power. Variance was determined based on the average of the two runs in each session, which is equivalent to one 10-minute scan. This was performed to minimize the effects of within-session variability and to reflect the variability observed in a longitudinal clinical study where multiple runs would not be acquired. The detection power was set to 80% (i.e., Z_1-β_ = 0.84) and the significance level was set to α = 0.05 based on a one-tailed *t*-test since the primary focus is on detecting regional perfusion deficits (i.e., Z_1- α_ = 1.645). A detectability map depicting the number of participants required to detect a 10% perfusion change between sessions was generated to visualize the estimated sample size for ROIs generated using the aal atlas.

To determine whether there were differences in CBF and ATT among the three ASL sequences, data were fit to a linear mixed model and an ANOVA used to test for significance. T-tests were used to assess between-group differences. For all tests *p* < 0.05 were considered significant. To assess the effect of day-to-day variations, the minimum detectable difference and reproducibility of SD-pCASL CBF were assessed using absolute (aCBF) and relative perfusion (rCBF), where relative perfusion was generated by intensity normalizing by the mean whole-brain CBF. Reliability was only calculated with aCBF, due to the reduction in between-subject variance after intensity normalization.

## 3 Results

### 3.1 Demographics and cognitive measures

Of the fourteen controls and ten patients recruited and screened for the current study, three patients and one control had missing follow-up data. The final sample of the test–retest study included 13 controls and 7 patients. Within-session scans were separated by approximately 30 min, while the average separation between imaging sessions was 26 ± 4 days. Data comparing ASL sequences included Hadamard-encoded data acquired in 9 controls and 8 patients. LowRes-pCASL and SD-pCASL data from the corresponding participants were included in this analysis. Demographic and clinical characteristics of all the participants are summarized in Table 1. As expected, healthy controls scored significantly higher on all cognitive tests (*p* < 0.05).

**Table 1:** Summary of demographics and cognitive measures.

	Patients		Controls	
Demographics				
Sex (M:F)		3:5		8 : 5
Age		68.8 ± 8.8		61.5 ± 9.6
Diagnosis		3 svFTD, 2 bvFTD, 2 nfPPA, 1 PSP		–

Cognitive Measures				
	N	Score	N	Score
ACE-III Total Score (American Version A)	8	54.8 ± 20	13	93.2 ± 3.5*§*
Mini-ACE Total Score (30)	8	13 ± 7.3	13	28.1 ± 2.2*§*
Attention (18)	8	15 ± 2.7	13	17.2 ± 1.5*§*
Memory (26)	8	10 ± 6.8	13	23.6 ± 2.8*§*
Fluency (14)	8	4.6 ± 3.4	13	11.9 ± 2.3*§*
Language (26)	8	12.6 ± 8.4	13	25.5 ± 1.1*§*
Visuospatial (16)	8	12.5 ± 2.1	13	15 ± 1*§*
Boston Naming (15)	8	4.3 ± 6.1	11	13.8 ± 1.6*§*
Geriatric Depression Scale (Short Form; 15)	8	3.8 ± 2.3	10	0.8 ± 1*§*

Caregiver Measures				
Neuropsychiatric Inventory Total Score (1 4 4)	7	11.6 ± 16	–	–
FBI Total Score (72)	8	22.1 ± 14.1	–	–
Cornell (38)	7	6.9 ± 4.7	–	–
Cambridge Behavioural Inventory Revised (1 8 0)	8	41.4 ± 18.7	–	–

Values are expressed as the mean ± standard deviation.

Values in parenthesis represent the maximum score for each test.

T-tests were conducted to test for differences in cognitive measures among patients and controls.

Statistical significance (p < 0.05) is indicated by §.

### 3.2 Test-Retest reproducibility of single delay pCASL

**Fig. 1:**
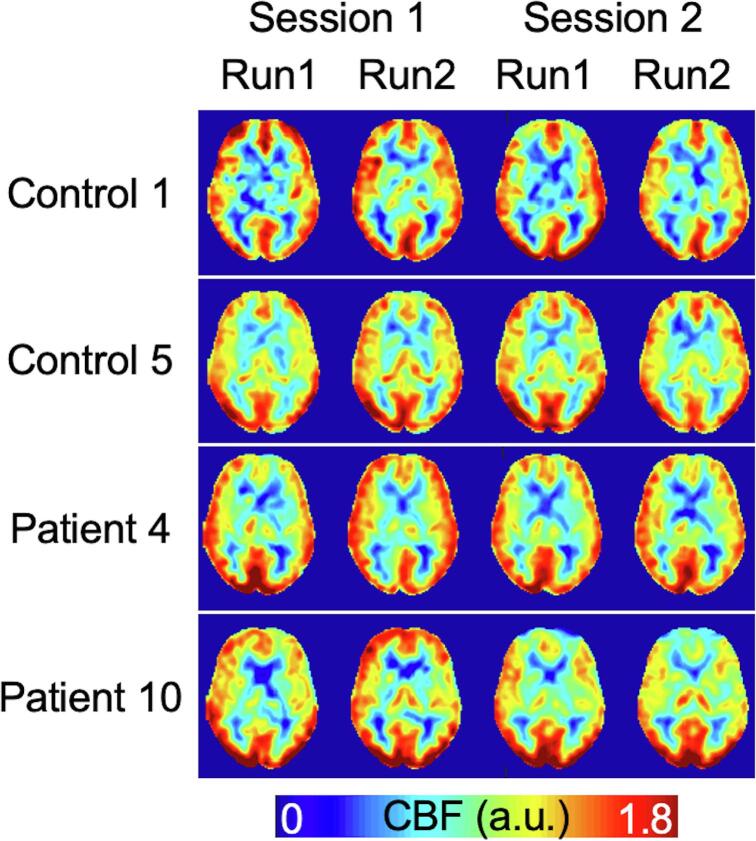
Example control and patient perfusion maps (in relative units) for each session and run.

Average grey-matter perfusion across all sessions was 68.6 ± 1.7 ml/100 g/min in controls and 65.2 ± 1.72 ml/100 g/min in patients. Representative perfusion images from example control and patient participants for the two sessions are shown in Fig. 1. Perfusion maps were scaled to a common range for display purposes. Perfusion maps showed the expected contrast between grey and white matter. While overall there was good agreement within and between sessions, there were noticeable differences in regional perfusion between sessions in some participants (e.g., reduced frontal perfusion for patient 10 during session 2). To a lesser extent, this phenomenon was also evident in control 1, session 2.

#### 3.2.1 Voxel-by-voxel variability

**Fig. 2:**
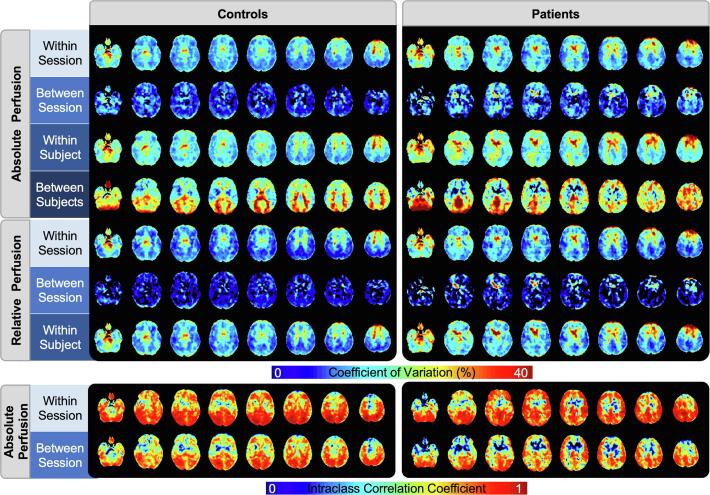
Coefficient of variation and intraclass correlation coefficient maps in patients and controls within-session, between-sessions, within-subject, and between-subjects. Both measures of variability were calculated for absolute CBF, but only the coefficient of variation for relative CBF.

Control and patient CV maps were similar, with both showing increased variance in white matter, cerebrospinal fluid, and regions proximal to the brain’s feeding vessels (Fig. 2). Following intensity normalization, there was a global decrease in grey-matter CV in both controls and patients; however, regions of high variability in white matter and cerebral spinal fluid remained. In controls, between-subject variability was higher in white matter, ventricles, and the posterior regions of the brain. Although between-subject reproducibility in grey-matter were within a similar range, (21.4% in controls and 25.3% in patients), in patients, distinct regions of increased variability including the superior frontal gyrus, cerebellum, brainstem, and the left intra-calcarine cortex were apparent. For both patients and controls, good-to-excellent reliability was determined for the within-session comparison, whereas fair-to-good reliability was achieved between sessions. For all comparisons, participants showed lower reliability in the striatum relative to other regions. In patients, a clear increase in grey-matter reliability relative to white matter was observed, especially between sessions.

Average voxel-by-voxel within and between-session variability in grey-matter aCBF were comparable for patients (within-session: 16%, between-session: 10.8%) and controls (within-session: 13.9%, between-session: 8.3%). Within-subjects variability in grey-matter was 19.2% and 16.2% in patients and controls, respectively. After intensity normalization, there was a small decrease in within-session variability (controls: 12.3%, patients: 14.8%), whereas between-session CV decreased to a greater extent (controls: 6%, patients: 8.5%). The corresponding within-subject variance were 16.3% and 13.9% in patients and controls, respectively. Good reliability among same-day scans in both patients (ICC_within_ = 0.73) and controls (ICC_within_ = 0.71) was found. Reliability was also good between sessions; however, there was a moderate decrease for both patients (ICC_between_ = 0.62) and controls (ICC_between_ = 0.62).

#### 3.2.2 Variability in FTD-specific ROIs

**Fig. 3:**
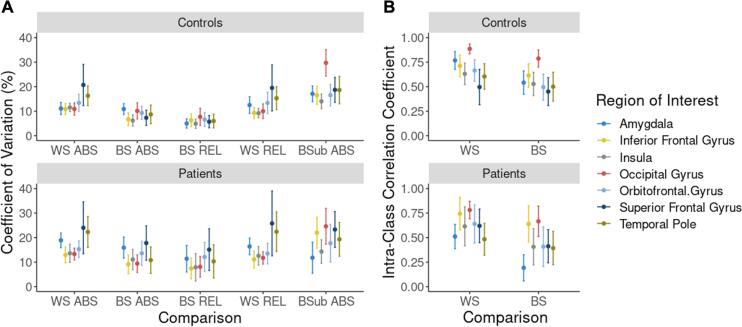
(A) Within-session (WS), between-session (BS), and between-subjects (BSub) reproducibility measured using absolute (ABS) and relative (REL) perfusion for (top) Controls and (bottom) Patients. (B) Reliability of within-session and between-session perfusion using absolute perfusion in (top) Controls and (bottom) Patients.

Across FTD-specific ROIs, there were no differences in perfusion between sessions or runs. Average reliability and reproducibility in FTD-specific ROIs are summarized in Fig. 3. The superior frontal gyrus and temporal pole showed a high amount of variability (CV > 15%) in patients and controls, whereas the orbitofrontal gyrus and amygdala showed high variability in patients only. Between sessions, CV was higher in the superior frontal gyrus and amygdala in patients. Intensity normalization resulted in a significant reduction in between-session CV (*p* < 0.05). With both aCBF and rCBF, within-session CV was significantly higher than between-session CV (*p* < 0.05). Within-session reliability was fair to excellent in both patients (range: 0.48–0.78) and controls (range: 0.5–0.89). Between sessions, there was a significant reduction in reliability for patients and controls; the majority of regions showed fair reliability; however, as indicated by ICC_between_ < 0.4, in patients reliability in the amygdala and temporal pole was poor.

#### 3.2.3 Detectability

**Fig. 4:**
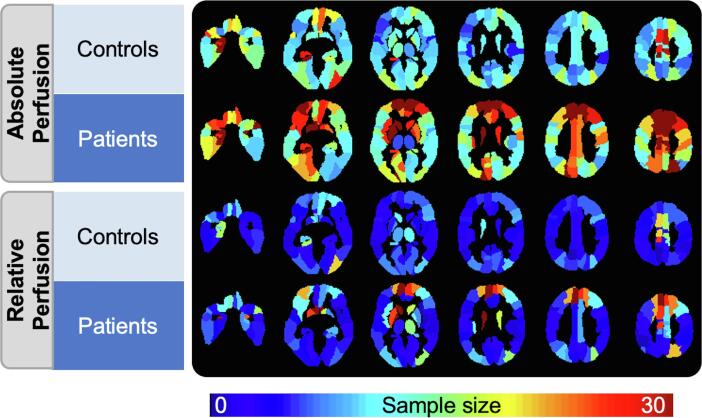
Detectability maps indicating the number of participants required to detect a 10% perfusion change within ROIs.

Detectability maps depicting estimated sample sizes required to detect a 10% decrease in perfusion between sessions using a 10-minute pCASL scan are shown in Fig. 4. Across ROIs, within-subject variance ranged between 5.1 and 14.5 ml/100 g/min for aCBF and 3.5 and 12.1 ml/100 g/min for rCBF. For FTD-specific ROIs, the number of participants required was significantly higher in patients (26 ± 14) relative to controls (13 ± 5) (*p* < 0.05). This estimate was based on averaging common ROIs on the left and right hemisphere. After intensity normalization, these values decreased to 10 ± 9 for patients and 5 ± 2 for controls (ns). Although intensity normalization improved regional detectability, as indicated by significant reduction in estimated sample sizes and the cooler colors in Fig. 4, in patients, the lowest sensitivity remained in the frontal lobe and sub-lobar regions.

### 3.3 Perfusion comparison in healthy controls

**Fig. 5:**
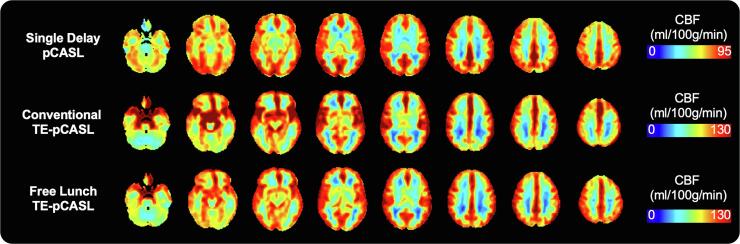
Perfusion averaged over healthy controls measured by (A) single delay pCASL, (B) free-lunch time-encoded pCASL and, (C) conventional time-encoded pCASL. Note, colour bar has been adjusted for each image for better visualization.

**Fig. 6:**
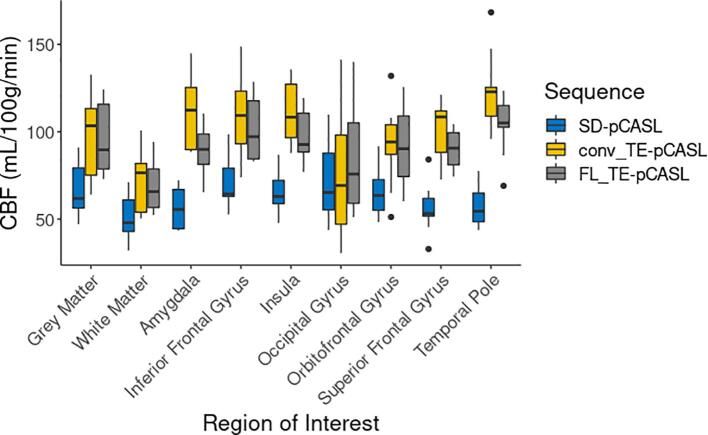
Comparison of SD-, FL_TE- and conv_TE-pCASL in grey and white-matter as well as regions commonly associated with FTD in healthy controls. Outliers are identified by black dots.

Perfusion maps averaged over healthy controls and generated by the three pCASL sequences are shown in Fig. 5. Each average contains an identical sample of controls. While all sequences show similarities in contrast and regional distribution between grey-and white matter perfusion, midbrain perfusion appears to be greater in FL_TE-pCASL and conv_TE-pCASL sequences. Average grey-matter CBF measured by SD-pCASL, FL_TE-pCASL and conv_TE-pCASL were: 67.7 ± 15, 95.6 ± 21.1, 96.7 ± 25 ml/100 g/min in controls. Perfusion averages within FTD-specific ROIs are shown in Fig. 6. Perfusion estimates by the Hadamard sequences had greater between-subject variability and were consistently higher than SD-pCASL estimates in all regions (*p* < 0.05) except for the occipital gyrus. Compared to the conv_TE-pCASL, FL_TE-pCASL was significantly lower in the amygdala, insula and temporal pole (*p* < 0.05).

### 3.4 Arterial transit time comparison in controls and patients

**Fig. 7:**
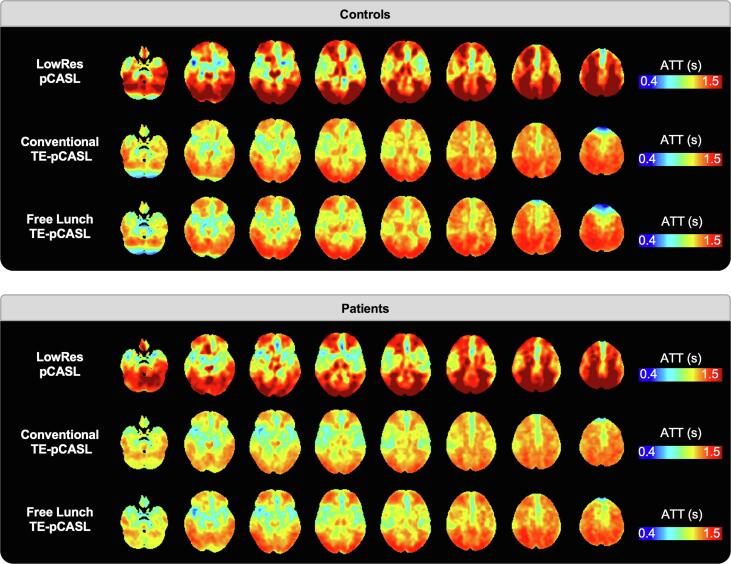
Average arterial transit time maps in patients and controls by LowRes-, FL_TE- and conv_TE-pCASL. Note, colour bar has been adjusted for each image for better visualization.

**Fig. 8:**
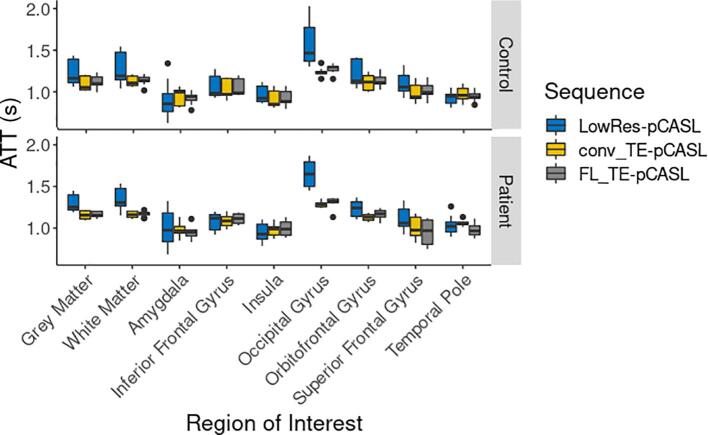
Comparison of LowRes-, conv_TE-, and FL_TE-pCASL, in grey and white-matter as well as regions commonly associated with FTD in controls (top) and patients (bottom). Outliers are identified by black dots.

Fig. 7 shows ATT maps generated using LowRes-pCASL, FL_TE-pCASL, and conv_TE-pCASL sequences generated for both controls and patients. All sequences show similar spatial patterns with shorter transit times near the centre of the major feeding arteries and increased transit times in the watershed regions. Grey-matter ATT measured by LowRes-pCASL, conv_TE-pCASL and, FL_TE-pCASL were 1.24 ± 0.16, 1.10 ± 0.08, and 1.12 ± 0.08 s in controls and 1.30 ± 0.11, 1.16 ± 0.05, and 1.17 ± 0.04 s in patients, respectively. For all sequences, ATT measured in patients were not significantly different from controls. Average ATT values in FTD specific ROIs are shown in Fig. 8. Among the three sequences, the only significant differences in transit times were in the occipital gyrus, orbitofrontal gyrus and superior frontal gyrus, where the LowRes-pCASL values were significantly higher than the corresponding conv_TE-pCASL and FL_TE-pCASL values. ATT CV maps were mostly homogeneous, with some non-specific spatial patterning (Supplementary Fig. 1). Average between-session CVs in grey-matter were 17.1 ± 5.9% and 15.2 ± 6.3% in controls and patients, respectively.

## 4 Discussion

Multiple studies have demonstrated the potential of ASL for assessing disease-driven perfusion changes that differentiate clinical populations as well as presymptomatic mutation carriers (Tosun et al., 2016, Mutsaerts et al., 2019, Anazodo et al., 2018). Considering that ASL is non-invasive and quantitative, it is well suited to longitudinal studies aimed at characterizing disease progression and evaluating treatment efficacy. Toward this goal, the current work focused on evaluating the reproducibility of an optimized ASL sequence for monitoring long-term changes in perfusion in FTD patients. As an initial evaluation, this study included patients who met the consensus criteria for probable FTD or PSP and age-matched controls. Imaging was performed in two sessions that were separated by four weeks – a period selected to minimize possible disease-related perfusion changes. Both within- and between-session variability was assessed to evaluate the impact of common sources of error associated with longitudinal studies including head repositioning and day-to-day fluctuations in CBF. Considering the impact of ATTs on CBF quantification, a second aim was to compare the performance of SD-pCASL and LowRes-pCASL, in which perfusion and ATT are measured separately, to Hadamard-encoded sequences that measure both parameters simultaneously. While the latter methods provide the ability to image faster with superior SNR, they are more sensitive to motion artifacts. To the best of our knowledge, this is the first study to perform this comparison in an older population. The main results of the study showed that (1) test–retest repeatability was similar in the patient group compared to controls, (2) variations in transit times were not a significant source of error with this patient population, and (3) perfusion imaging by Hadamard-encoded sequences yielded systematically higher CBF compared to SD-pCASL but produced similar transit-time measurements compared to LowRes-pCASL.

Since the role of ASL in assisting with the diagnosis of FTD subtypes is to detect spatial patterns of hypoperfusion, the current study primarily focused on characterizing variability on a voxel-by-voxel basis. This approach provided the ability to identify regions with greater variability, which could make it more challenging to detect perfusion changes in longitudinal studies. In general, within-subject reproducibility and reliability maps, shown in Fig. 2 and summarized in Fig. 3, for the patient and control groups were similar. Between-session grey-matter reproducibility and reliability for patients were similar to values for controls (CV = 10.8% vs 8.3%, ICC_between_ = 0.62 vs 0.62, respectively). Regions with the highest variability (the superior frontal gyrus and temporal pole) were common to both groups (Fig. 3), although the CV in dementia-specific ROIs were significantly lower for controls. Increased variability in the superior frontal gyrus and temporal pole are likely related to susceptibility artifacts due to brain-air interfaces, particularly in the patient population where there is greater brain atrophy (Zhao et al., 2017). Visual inspection of Fig. 2 revealed that in both patient and control participants, there was an imaging artifact in the sub-lobar region that is consistent with signal dephasing due to the pulsatile flow in the circle of Willis during the GRASE readout. In the patient group, its border spread into the amygdala region, explaining the increased variability in this region. While a segmented sequence was implemented to reduce the effects of T2 decay during the readout (Feinberg and Günther, 2009), it may be possible to further reduce this artifact by using a variable flip angle (Liang et al., 2013, Zhao et al., 2018). Between-subject reproducibility among patients showed increased CV in the occipital and posterior regions. While this increase could reflect the disease-driven heterogeneity in regional hypoperfusion, since these regions are not typically affected by in FTD and the related disorders, it more likely reflects individual differences due to the small sample size.

Both the accuracy and precision of ASL-CBF are affected by factors such as its inherently low SNR, subject motion, sensitivity to transit delays, and labeling efficiency (Chen et al., 2011). In addition to these within-session sources of error, factors that can degrade between-session reproducibility include repositioning errors and differences in resting perfusion between scanning sessions (Ssali et al., 2016). Efforts to minimize these sources of variability include conducting repeat imaging sessions around the same time of day (Parkes et al., 2004), having participants avoid substances known to affect CBF (e.g. caffeine) (Clement et al., 2018), and implementing a pre-processing pipeline, including ENABLE, to ensure the quality of the ASL images and good registration to the MNI template (Shirzadi et al., 2018, Mutsaerts et al., 2018). The similarity of perfusion maps separated by a month (Fig. 1) demonstrated the effectiveness of these approaches. Between-session variability in grey-matter showed good correlation (ICC_between_ > 0.6) and good reproducibility in both patients and controls (CV < 11%). With intensity normalization, there was approximately a 28 and 21% reduction in between-session CV in patients and controls respectively, highlighting the systemic effect of day-to-day fluctuations in resting perfusion (Fig. 2). Together, these results suggest that with good alignment of data between sessions, and careful control of perfusion modifiers, sources of between-session variability can be minimized. This is particularly relevant for clinical diagnosis and management of FTD given that perfusion changes are subtle.

ROI-based between-session reproducibility and reliability across grey-matter for patients (CV = 9.04%, ICC_between_ = 0.77) and controls (CV = 6.5%, ICC_between_ = 0.77) were within the range of previous studies of other causes of dementia. Kilroy et al assessed reproducibility and reliability of pCASL GRASE in a population of older healthy controls, and patients with MCI and Alzheimer’s disease (Kilroy et al., 2014). The authors reported a CV of 10.9% and an ICC_between_ of 0.707 among perfusion measurements separated by 4 weeks. Similar results were observed in different scan separations among young and older participants. Chen et al reported a CV of 8.5 ± 0.14% for data collected 1 week apart in young healthy participants (Chen et al., 2011); more recently, in a population of adult Latinx participants at risk for vascular disease, Jann et al. reported a CV of 7% and ICC_between_ of 0.84 (Jann et al., 2021). The finding that longitudinal variability of the current implementation of ASL is comparable to, and in some cases superior to previous studies, supports the potential of ASL as a sensitive marker of longitudinal perfusion changes.

Metrics of within-session reproducibility and reliability were similar in both patients and controls (Fig. 2). In grey-matter, patients and controls CVs were within roughly 14% of each other (i.e. 16% vs 13.8%, respectively) and correlations between repeat measurements were good (i.e. 0.73 vs 0.71). An unexpected finding was the reproducibility between sessions was greater than the reproducibility between runs in the same session. Within-session variance was 64 and 70% of the within-subject variance in patients and controls. After intensity normalization, these proportions were: 70 and 77% in the two groups, respectively. This suggests that even after minimizing between-session variance, within-session variance remained dominant. One possible explanation is that perfusion modifiers such as arousal and attention could have led to changes in global CBF between the two runs (Clement et al., 2018). As participants acclimatized to the scanner environment, cerebral perfusion could have decreased considering the two runs were separated by approximately 30 min. However, a repeated measures ANOVA confirmed that average grey-matter perfusion between runs were not significantly different. A more likely explanation is the inherently low SNR of the ASL sequence. Considering that the ASL signal used to calculate perfusion is on the order of 1% (Alsop et al., 2015), several tag-control pairs are typically acquired to improve SNR. In the current study, 8 tag-control pairs were collected in each run to keep the scan time around 5 min, which is typical for clinical studies. However, the unexpectedly poor within-session reproducibility indicates that more averages should be acquired. In order to more accurately characterize perfusion, we recommend a 10-minute SD-pCASL scan.

As a means of visualizing the impact of between-session variability on tracking longitudinal changes in regional CBF, detectability maps were created to show the predicted sample size that would be required to detect a 10% perfusion change in individual anatomical regions (Fig. 4). In light of the unexpectedly high within-session variability, data from the two runs in each session were combined and within-subject (i.e. between-session) variance was estimated based on the two resulting 10-minute SD-pCASL scans. Focusing on FTD-specific ROIs, the predicted sample sizes required for patients (aCBF = 26 ± 14, rCBF = 10 ± 9) were generally larger than those required for controls (aCBF = 13 ± 5, rCBF = 5 ± 2). However, this difference only reached statistical significance for absolute CBF. For both groups, a greater number of participants were predicted for ROIs in the frontal and occipital lobes, particularly near watershed regions (Fig. 4). These findings could be related to differences in labeling efficiency between sessions. In an effort to maximize labeling efficiency, a time-of-flight image was used to locate the ideal location for the ASL labeling plane during the first session. Since identical parameters were repeated during the second session, it is possible that the labeling location was not ideal, which could influence the image contrast considering the tortuosity of feeding brain vessels increases with age (Lee et al., 2009, Qiu et al., 2010). This variability was observed in both patients and controls as evident in Fig. 1 in which control 1 and patient 10 exhibited reduced frontal perfusion during the second imaging session. Nevertheless, the prediction that roughly 10 participants would be required to detect a 10% perfusion change in regions relevant to FTD indicates that with careful parameter selection, the current implementation of ASL has the sensitivity to detect longitudinal perfusion changes with relatively small sample sizes. This finding is promising for clinical studies given that FTD is relatively rare (Staffaroni et al., 2019a), which can make recruitment of large number of patients challenging.

Since the prevalence of cerebrovascular disease among patients with FTD is low (Toledo et al., 2013, De Reuck et al., 2012, McKhann, 2001), variation in transit times between the different subtypes was not expected, and therefore, ATT data were averaged across all patient participants. Visual inspection of the ATT maps averaged across patients shows strong resemblance to the corresponding maps generated from the controls (Fig. 7). In addition, average whole-brain ATT values for controls and patients were not significantly different. Likewise, between-session whole-brain CV for patients (15.2 ± 6.3%) and controls (17.1 ± 5.9%) were similar, and the CV maps were homogeneous (S Fig. 1), indicating no regional effects. In contrast to patients with Alzheimer’s disease, for whom cerebrovascular dysfunction can result in compromised perfusion (Alsop et al., 2000, De Jong et al., 2019, Mak et al., 2012), there is limited evidence of vascular degeneration in FTD patients beyond that typically attributed to age (De Reuck et al., 2012). In the absence of a priori knowledge, the current study used a PLD of 2 s, based on the recommendations of the ASL white paper (Alsop et al., 2015). Although some studies have shown increased sensitivity by correcting for transit times (Cohen et al., 2020), on average, less than 4.2 ± 4.4% of transit times measured in the current study were greater than the selected PLD. When ATT values greater than 2.3 s were considered, this value dropped to 0.8 ± 0.9%. These results are in agreement with Dai et al. who reported that a PLD between 2 and 2.3 s is sufficient for imaging elderly cohorts (Dai et al., 2017). Given that intensity normalization further diminishes the need for ATT correction (Dai et al., 2017) and yields greater reproducibility and reliability (Ssali et al., 2016), a PLD of 2 s is sufficient for SD-pCASL imaging of FTD patients. This is particularly encouraging for studies focused on assessing presymptomatic perfusion changes considering the effects of ATT should be even more muted due to the younger age of the participants.

While single delay pCASL with a 3D readout is currently recommended for ASL perfusion imaging (Alsop et al., 2015), novel Hadamard encoded approaches are gaining interest due to their ability to image both CBF and ATT in a similar amount of time with good spatial resolution and SNR (Samson-himmelstjerna et al., 2016, Dai et al., 2013, van Osch et al., 2018). Of note, FL_TE-pCASL offers the benefit of acquiring both perfusion and transit time images with similar PLD and LD as the single delay sequence, but without an increase in scan time (Wells et al., 2010, Günther, 2006). While visual inspection revealed greater agreement between FL_TE-pCASL and SD-pCASL, both Hadamard-encoded sequences showed increased contrast in the basal ganglia and insula, compared to SD-pCASL (Fig. 5). This difference could be attributed to z-direction blurring, a well-documented artifact associated with GRASE readout, as well as differences in labeling parameters between the sequences (Woods et al., 2020). Hadamard-based perfusion were not significantly different from SD-pCASL in the occipital gyrus. This reduced perfusion is in line with the superior posterior watershed region that experiences longer ATTs and, furthermore, inspection of perfusion maps revealed hyperperfusion where the vessels enter the cerebrum (e.g. the circle of Willis), particularly with conv_TE-pCASL. Hadamard encoded sequences produced significantly higher CBF estimates relative to SD-pCASL (Fig. 6). To date, few studies have performed a head-to-head comparison of these sequences and no studies in older populations. A recent study demonstrated that CBF by conv_TE-pCASL was 22% higher perfusion in a population of young healthy participants (Guo et al., 2018). Given that internal consistency is critical for longitudinal imaging, the good reproducibility of conv_TE-pCASL (CV = 10.5%, ICC_between_ = 0.77) over a 45 day period reported by Cohen et al., highlights the potential for these novel sequences (Cohen et al., 2020). Furthermore, as demonstrated by the results of this study, relative perfusion provides a stable estimate of perfusion over time, making this systematic offset less concerning.

There are a number of limitations with the current study. First, the estimates of variability were conducted with small sample sizes. Despite this, the results among patients and controls were similar to each other as well as to previous studies. Considering that different subtypes may have differing degrees of regional variability, future studies could investigate subtype-specific sensitivities. Another consideration was the use of global CBF for normalizing the perfusion images. Reference regions need to be selected carefully since errors can be introduced if there are regional perfusion deficits that alter global CBF (Borghammer et al., 2008). While the cerebellum is often used as a reference region, it is not always within the ASL FOV, and furthermore, studies have identified cerebellar atrophy in some patients with FTD (Schmahmann, 2016, Gellersen, 2017). Since there was no difference in global perfusion between patients and controls, global CBF was chosen as a suitable reference region. The low spatial resolution compounded with regional brain atrophy in patient participants can lead to reduced perfusion due to partial volume errors. While the current study did not investigate the influence of partial volume errors, previous work demonstrated that similar hypoperfusion patterns were detected without partial volume correction applied (Dolui et al., 2020). More importantly, a between-session delay of 4 weeks was chosen to avoid any atrophy-driven perfusion changes between sessions. Finally, although efforts were made to select optimal labeling and bolus/sub-bolus durations among the ASL sequences being compared, it is evident that the Hadamard encoded sequences could have been further optimized (e.g. increasing the sub-bolus durations and PLD) to reduce the effects of vascular signal (Woods et al., 2020).

## 5 Conclusion

The results of the current study indicate that SD-pCASL with the appropriate labeling parameters is a promising approach for assessing longitudinal changes in CBF associated with FTD. With the current implementation, it was predicted that ASL can reliably detect changes in perfusion as small as 10% with an estimated sample size of 10 patients with relative perfusion. Agreement of longitudinal measures of CBF and ATT were similar in patients and controls, indicating that there was no additional source of variability with FTD patients compared to age-matched controls. Relative to Hadamard-encoded sequences, SD-pCASL showed better grey-to-white matter contrast; however, Hadamard-encoded ASL showed better contrast in the deep-brain regions. While the current study assessed the variability of perfusion measurements within-subject, another important aspect in the diagnosis of FTD is the ability to assess differences between patients and controls. Future work could evaluate the sensitivity of ASL for detecting disease-driven perfusion changes by direct comparison to the gold standard, PET with radiolabeled water (Ssali et al., 2018). Additionally, toward the ultimate goal of assessing longitudinal perfusion changes, methodologies described in the current study could be implemented in large multi-centre studies to gain greater insight into the potential clinical role of ASL in diagnosis and management of FTD.

## CRediT authorship contribution statement

**Tracy Ssali:** Conceptualization, Data curation, Formal analysis, Investigation, Methodology, Project administration, Resources, Software, Validation, Visualization, Writing – original draft, Writing - review & editing. **Udunna C. Anazodo:** Investigation, Methodology, Resources, Writing - review & editing. **Lucas Narciso:** Investigation, Methodology, Writing - review & editing. **Linshan Liu:** Investigation, Writing - review & editing. **Sarah Jesso:** Investigation, Resources, Writing - review & editing. **Lauryn Richardson:** Investigation, Resources, Writing – review & editing. **Matthias Günther:** Resources, Software, Writing - review & editing. **Simon Konstandin:** Resources, Software, Writing - review & editing. **Klaus Eickel:** Resources, Software, Writing - review & editing. **Frank Prato:** Supervision, Writing - review & editing. **Elizabeth Finger:** Resources, Supervision, Writing - review & editing. **Keith St. Lawrence:** Conceptualization, Formal analysis, Funding acquisition, Methodology, Project administration, Resources, Supervision, Validation, Writing - review & editing.

## References

[b0005] Alexopoulos G.S., Abrams R.C., Young R.C., Shamoian C.A. (1988). Cornell scale for depression in dementia. Soc. Biol. Psychiatry.

[b0010] Alsop D.C. (2015). Recommended implementation of arterial spin-labeled perfusion MRI for clinical applications: a consensus of the ISMRM perfusion study group and the European consortium for ASL in dementia. Magn. Reson. Med..

[b0015] Alsop D.C., Detre J.A., Grossman M. (2000). Assessment of cerebral blood flow in Alzheimer’s disease by spin-labeled magnetic resonance imaging. Ann. Neurol..

[b0020] Anazodo U.C. (2018). Using simultaneous PET/MRI to compare the accuracy of diagnosing frontotemporal dementia by arterial spin labelling MRI and FDG-PET. NeuroImage Clin..

[b0025] Ashburner J. (2012). A history. Neuroimage.

[b0030] Borghammer P., Jonsdottir K.Y., Cumming P., Ostergaard K., Vang K., Ashkanian M., Vafaee M., Iversen P., Gjedde A. (2008). Normalization in PET group comparison studies–the importance of a valid reference region. Neuroimage.

[b0035] Buxton R.B. (2005). Quantifying CBF with arterial spin labeling. J. Magn. Reson. Imaging.

[b0040] Chan D., Fox N.C., Jenkins R., Scahill R.I., Crum W.R., Rossor M.N. (2001). Rates of global and regional cerebral atrophy in AD and frontotemporal dementia. Neurology.

[b0045] Chappell M.A., Groves A.R., Whitcher B., Woolrich M.W. (2009). Variational bayesian inference for a nonlinear forward model. IEEE Trans. Signal Process..

[b0050] Chen Y., Wang D.J.J., Detre J.A. (2011). Test-retest reliability of arterial spin labeling with common labeling strategies. J. Magn. Reson. Imag..

[b0055] Cicchetti D.V., Sparrow S.A. (1981). Developing criteria for establishing interrater reliability of specific items: applications to assessment of adaptive behavior. Am. J. Ment. Defic..

[b0060] Clement P. (2018). Variability of physiological brain perfusion in healthy subjects – A systematic review of modifiers. Considerations for multi-center ASL studies. J. Cereb. Blood Flow Metab..

[b0065] Cohen A.D., Agarwal M., Jagra A.S., Nencka A.S., Meier T.B., Lebel R.M., McCrea M.A., Wang Y. (2020). Longitudinal reproducibility of MR perfusion using 3D pseudocontinuous arterial spin labeling with hadamard. Encoded Multiple Postlabel. Delays.

[b0070] Cummings J.L. (1994). The neuropsychiatric inventory: comprehensive assessment of psychopathology in dementia. Neurology.

[b0075] Dai W. (2017). Effects of arterial transit delay on cerebral blood flow quantification using arterial spin labeling in an elderly cohort. J. Magn. Reson. Imaging.

[b0080] Dai W., Garcia D., de Bazelaire C., Alsop D.C. (2008). Continuous flow-driven inversion for arterial spin labeling using pulsed radio frequency and gradient fields. Magn. Reson. Med..

[b0085] Dai W., Shankaranarayanan A., Alsop D.C. (2013). Volumetric measurement of perfusion and arterial transit delay using hadamard encoded continuous arterial spin labeling. Magn. Reson. Med..

[b0090] De Jong D.L.K., de Heus R.A.A., Rijpma A., Donders R., Olde Rikkert M.G.M., Günther M., Lawlor B.A., van Osch M.J.P., Claassen J.A.H.R. (2019). Effects of nilvadipine on cerebral blood flow in patients with alzheimer disease: a randomized trial. Hypertension.

[b0095] De Reuck J.L. (2012). Cerebrovascular lesions in patients with frontotemporal lobar degeneration: A neuropathological study. Neurodegener. Dis..

[b0100] Diehl-Schmid J. (2007). Decline of cerebral glucose metabolism in frontotemporal dementia: a longitudinal 18F-FDG-PET-study. Neurobiol. Aging.

[b0105] Dolui S., Li Z., Nasrallah I.M., Detre J.A., Wolk D.A. (2020). Arterial spin labeling versus 18F-FDG-PET to identify mild cognitive impairment. NeuroImage Clin..

[b0110] Dopper E.G.P. (2016). Cerebral blood flow in presymptomatic MAPT and GRN mutation carriers: a longitudinal arterial spin labeling study. NeuroImage Clin..

[b0115] Farkas E., Luiten P.G.M. (2001). Cerebral microvascular pathology in aging and Alzheimer’s disease. Progr. Neurobiol..

[b0120] Feinberg D.A., Günther M. (2009). Cerebral blood flow imaging with 3D GRASE ASL sequence increases SNR and Shortens acquisition time. MAGNETOM Flash.

[b0125] Finger, E. C. Frontotemporal Dementias. Contin. Lifelong Learn. Neurol. 22, 464–489 (2016). 10.1212/CON.0000000000000300 PMC5390934 27042904

[b0130] Gellersen H.M. (2017). Cerebellar atrophy in neurodegeneration – a meta-analysis. J. Neurol. Neurosurg. Psychiatry.

[b0135] Gorno-Tempini M.L., Hillis A.E., Weintraub S., Kertesz A., Mendez M., Cappa S.F., Ogar J.M., Rohrer J.D., Black S., Boeve B.F., Manes F., Dronkers N.F., Vandenberghe R., Rascovsky K., Patterson K., Miller B.L., Knopman D.S., Hodges J.R., Mesulam M.M., Grossman M. (2011). Classification of primary progressive aphasia and its variants. Neurology.

[b0140] Groves A.R., Chappell M.A., Woolrich M.W. (2009). Combined spatial and non-spatial prior for inference on MRI time-series. Neuroimage.

[b0145] Günther M. (2006). Efficient visualization of vascular territories in the human brain by cycled arterial spin labeling MRI. Magn. Reson. Med..

[b0150] Günther M., Oshio K., Feinberg D.A. (2005). Single-shot 3D imaging techniques improve arterial spin labeling perfusion measurements. Magn. Reson. Med..

[b0155] Guo J., Holdsworth S.J., Fan A.P., Lebel M.R., Zun Z., Shankaranarayanan A., Zaharchuk G. (2018). Comparing accuracy and reproducibility of sequential and Hadamard-encoded multidelay pseudocontinuous arterial spin labeling for measuring cerebral blood flow and arterial transit time in healthy subjects: a simulation and in vivo study. J. Magn. Reson. Imaging.

[b0160] Herscovitch P., Raichle M.E. (1985). What is the correct value for the brain–blood partition coefficient for water?. J. Cereb. Blood Flow Metab..

[b0165] Höglinger G.U. (2017). Clinical diagnosis of progressive supranuclear palsy: the movement disorder society criteria. Mov. Disord..

[b0170] Hox, J. J., Moerbeek, M. & Schoot, R. van de. Multilevel analysis : techniques and applications / Joop J. Hox, Mirjam Moerbeek, Rens van de Schoot. (Routledge, 2018).

[b0175] Iturria-Medina Y., Sotero R.C., Toussaint P.J., Mateos-Pérez J.M., Evans A.C. (2016). Early role of vascular dysregulation on late-onset Alzheimer’s disease based on multifactorial data-driven analysis. Nat. Commun..

[b0180] Jann K. (2021). Evaluation of cerebral blood flow measured by 3D PCASL as biomarker of vascular cognitive impairment and dementia (VCID) in a cohort of elderly latinx subjects at risk of small vessel disease. Front. Neurosci..

[b0185] Jenkinson M., Bannister P., Brady M., Smith S. (2002). Improved optimization for the robust and accurate linear registration and motion correction of brain images. Neuroimage.

[b0190] Jenkinson, M., Beckmann, C. F., Behrens, T. E. J., Woolrich, M. W. & Smith, S. M. FSL. Neuroimage 62, 782–90 (2012). 10.1016/j.neuroimage.2011.09.015 21979382

[b0195] Jenkinson M., Smith S. (2001). A global optimisation method for robust affine registration of brain images. Med. Image Anal..

[b0200] Jiskoot, L. C. et al. Longitudinal multimodal MRI as prognostic and diagnostic biomarker in presymptomatic familial frontotemporal dementia. Brain 142, 193–208 (2019). 10.1093/brain/awy288 PMC6308313 30508042

[b0205] Kaplan E., Goodglass H., Weintraub S. (1983).

[b0210] Kertesz A., Nadkarni N., Davidson W., Thomas A.W. (2000). The frontal behavioral inventory in the differential diagnosis of frontotemporal dementia. J. Int. Neuropsychol. Soc..

[b0215] Kilroy E. (2014). Reliability of two-dimensional and three-dimensional pseudo-continuous arterial spin labeling perfusion MRI in elderly populations: comparison with 15o-water positron emission tomography. J. Magn. Reson. Imaging.

[b0220] Lee C. (2009). Imaging cerebral blood flow in the cognitively normal aging brain with arterial Spin labeling: Implications for imaging of neurodegenerative disease. J. Neuroimaging..

[b0225] Liang X., Connelly A., Calamante F. (2013). Improved partial volume correction for single inversion time arterial spin labeling data. Magn. Reson. Med..

[b0230] Logroscino G. (2019). Promising therapies for the treatment of frontotemporal dementia clinical phenotypes: from symptomatic to disease-modifying drugs. Expert Opin. Pharmacother..

[b0235] Mak H.K.F. (2012). Quantitative assessment of cerebral hemodynamic parameters by QUASAR arterial spin labeling in alzheimer’s disease and cognitively normal elderly adults at 3-Tesla. J. Alzheimer’s Dis..

[b0240] McKhann G.M. (2001). Clinical and pathological diagnosis of frontotemporal dementia. Arch. Neurol..

[b0245] Mioshi E., Dawson K., Mitchell J., Arnold R., Hodges J.R. (2006). The addenbrooke’s cognitive examination revised (ACE-R): a brief cognitive test battery for dementia screening. Int. J. Geriatr. Psychiatry.

[b0250] Murphy K. (2011). Pulsed arterial spin labeling perfusion imaging at 3 T: estimating the number of subjects required in common designs of clinical trials. Magn. Reson. Imaging.

[b0255] Mutsaerts, H. J. M. M. et al. Cerebral perfusion changes in presymptomatic genetic frontotemporal dementia: a GENFI study. Brain 142, 1108–1120 (2019). 10.1093/brain/awz039 PMC6439322 30847466

[b0260] Mutsaerts, H. J. M. M. et al. Comparison of Arterial Spin Labeling Registration Strategies in the Multi-center GENetic Frontotemporal dementia Initiative (GENFI). 131–140 (2017) doi:10.1002/jmri.25751. 10.1002/jmri.25751 PMC6485386 28480617

[b0265] Olm C.A. (2016). Arterial spin labeling perfusion predicts longitudinal decline in semantic variant primary progressive aphasia. J. Neurol..

[b0270] Parkes L.M., Rashid W., Chard D.T., Tofts P.S. (2004). Normal cerebral perfusion measurements using arterial spin labeling: reproducibility, stability, and age and gender effects. Magn. Reson. Med..

[b0275] Piguet O., Hornberger M., Mioshi E., Hodges J.R. (2011). Behavioural-variant frontotemporal dementia: Diagnosis, clinical staging, and management. Lancet Neurol..

[b0280] Qiu M. (2010). Arterial transit time effects in pulsed arterial spin labeling CBF mapping: Insight from a PET and MR study in normal human subjects. Magn. Reson. Med..

[b0285] Rajagopalan V., Pioro E.P. (2019). Longitudinal 18F-FDG PET and MRI reveal evolving imaging pathology that corresponds to disease progression in a patient with ALS-FTD. Front. Neurol..

[b0290] Rascovsky, K. et al. Sensitivity of revised diagnostic criteria for the behavioural variant of frontotemporal dementia. Brain 134, 2456–2477 (2011). 10.1093/brain/awr179 PMC3170532 21810890

[b0295] Samson-himmelstjerna, F. Von, Madai, V. I., Sobesky, J. & Guenther, M. Walsh-Ordered Hadamard Time-Encoded Pseudocontinuous ASL (WH pCASL). 1824, 1814–1824 (2016). 10.1002/mrm.26078 26714671

[b0300] Schmahmann J.D. (2016). Cerebellum in Alzheimer’s disease and frontotemporal dementia: not a silent bystander. Brain.

[b0305] Schuster C., Elamin M., Hardiman O., Bede P. (2015). Presymptomatic and longitudinal neuroimaging in neurodegeneration-from snapshots to motion picture: a systematic review. J. Neurol. Neurosurg. Psychiatry.

[b0310] Seelaar H., Rohrer J.D., Pijnenburg Y.A.L., Fox N.C., Van Swieten J.C. (2011). Clinical, genetic and pathological heterogeneity of frontotemporal dementia: a review. J. Neurol. Neurosurg. Psychiatry.

[b0315] Shavelson, R. J. & Webb, N. M. Generalizability Theory: A Primer. vol. 1 (Sage Publications, 1991).

[b0320] Sheikh J.I., Yesavage J.A. (1986). Geriatric depression scale (GDS): recent evidence and development of a shorter version. Clin. Gerontol. J. Aging Ment. Heal..

[b0325] Shirzadi Z. (2018). Enhancement of automated blood flow estimates (ENABLE) from arterial spin-labeled MRI. J. Magn. Reson. Imaging.

[b0330] Shrout P.E., Fleiss J.L. (1979). Intraclass correlations: uses in assessing rater reliability. Psychol. Bull..

[b0335] Smith S.M. (2004). Overview of fMRI analysis. Br. J. Radiol..

[b0340] Smith S.M., Zhang Y., Jenkinson M., Chen J., Matthews P.M., Federico A., De Stefano N. (2002). Accurate, robust, and automated longitudinal and cross-sectional brain change analysis. Neuroimage.

[b0345] Ssali T., Anazodo U.C., Bureau Y., MacIntosh B.J., Günther M., St. Lawrence K., Chen K. (2016). Mapping long-term functional changes in cerebral blood flow by arterial spin labeling. PLoS One.

[b0350] Ssali T., Anazodo U.C., Thiessen J.D., Prato F.S., St. Lawrence K. (2018). A non-invasive method for quantifying cerebral blood flow by hybrid PET/MR. J. Nucl. Med..

[b0355] Staffaroni A.M., Cobigo Y., Elahi F.M., Casaletto K.B., Walters S.M., Wolf A., Lindbergh C.A., Rosen H.J., Kramer J.H. (2019). A longitudinal characterization of perfusion in the aging brain and associations with cognition and neural structure. Hum. Brain Mapp..

[b0360] Staffaroni, A. M. et al. Longitudinal multimodal imaging and clinical endpoints for frontotemporal dementia clinical trials. Brain 142, 443–459 (2019). 10.1093/brain/awy319 PMC6351779 30698757

[b0365] Teeuwisse W.M., Schmid S., Ghariq E., Veer I.M., Van Osch M.J.P. (2014). Time-encoded pseudocontinuous arterial spin labeling: basic properties and timing strategies for human applications. Magn. Reson. Med..

[b0370] Toledo, J. B. et al. Contribution of cerebrovascular disease in autopsy confirmed neurodegenerative disease cases in the National Alzheimer’s Coordinating Centre. Brain 136, 2697–2706 (2013). 10.1093/brain/awt188 PMC3858112 23842566

[b0375] Tosun D. (2016). Diagnostic utility of ASL-MRI and FDG-PET in the behavioral variant of FTD and AD. Ann. Clin. Transl. Neurol..

[b0380] van Osch M.JP., Teeuwisse W.M., Chen Z., Suzuki Y., Helle M., Schmid S. (2018). Advances in arterial spin labelling MRI methods for measuring perfusion and collateral flow. J. Cereb. Blood Flow Metab..

[b0385] Vanderwal T., Kelly C., Eilbott J., Mayes L.C., Castellanos F.X. (2015). Inscapes: a movie paradigm to improve compliance in functional magnetic resonance imaging. Neuroimage.

[b0390] Verfaillie S.C.J. (2015). Cerebral perfusion and glucose metabolism in Alzheimer’s disease and frontotemporal dementia: two sides of the same coin?. Eur. Radiol..

[b0395] Wansapura J.P., Holland S.K., Dunn R.S., Ball W.S. (1999). NMR relaxation times in the human brain at 3.0 tesla. J. Magn. Reson. Imaging.

[b0400] Warren, J. D., Rohrer, J. D. & Rossor, M. N. Frontotemporal dementia. BMJ 347, 1–9 (2013). 10.1136/bmj.f4827 PMC3735339 23920254

[b0405] Wedderburn C. (2008). The utility of the Cambridge Behavioural Inventory in neurodegenerative disease. J. Neurol. Neurosurg. Psychiatry.

[b0410] Wells J.A., Lythgoe M.F., Gadian D.G., Ordidge R.J., Thomas D.L. (2010). In vivo hadamard encoded continuous arterial spin labeling (H-CASL). Magn. Reson. Med..

[b0415] Whitwell J.L. (2015). Brain atrophy over time in genetic and sporadic frontotemporal dementia: A study of 198 serial magnetic resonance images. Eur. J. Neurol..

[b0420] Whitwell J.L., Josephs K.A. (2012). Recent advances in the imaging of frontotemporal dementia. Curr. Neurol. Neurosci. Rep..

[b0425] Whitwell J.L., Jack C.R., Boeve B.F., Senjem M.L., Baker M., Rademakers R., Ivnik R.J., Knopman D.S., Wszolek Z.K., Petersen R.C., Josephs K.A. (2009). Voxel-based morphometry patterns of atrophy in FTLD with mutations in MAPT or PGRN. Neurology.

[b0430] Woods J.G., Chappell M.A., Okell T.W. (2020). Designing and comparing optimized pseudo-continuous arterial spin labeling protocols for measurement of cerebral blood flow. Neuroimage.

[b0435] Xu G. (2010). Reliability and precision of pseudo-continuous arterial spin labeling perfusion MRI on 3.0 T and comparison with 15O-water PET in elderly subjects at risk for Alzheimer’s disease. NMR Biomed..

[b0440] Zhang X. (2013). In vivo blood T1 measurements at 1.5 T, 3 T, and 7 T. Magn. Reson. Med..

[b0445] Zhao L., Chang C.D., Alsop D.C. (2018). Controlling T 2 blurring in 3D RARE arterial spin labeling acquisition through optimal combination of variable flip angles and k-space filtering. Magn. Reson. Med..

[b0450] Zhao M.Y., Mezue M., Segerdahl A.R., Okell T.W., Tracey I., Xiao Y., Chappell M.A. (2017). A systematic study of the sensitivity of partial volume correction methods for the quantification of perfusion from pseudo-continuous arterial spin labeling MRI. Neuroimage.

