# Supplementary material for: Sensitivity of Arterial Spin Labeling for Characterization of Longitudinal Perfusion Changes in Frontotemporal Dementia and Related Disorders
Source: Neuroimage Clin. 2021 Oct 7;35:102853. doi: 10.1016/j.nicl.2021.102853 (PMC9421452; doi:10.1016/j.nicl.2021.102853)
Supplement: Supplementary data 1 [file mmc1.docx]

# Sensitivity of Arterial Spin Labeling for Characterization of Longitudinal Perfusion Changes in Frontotemporal Dementia and Related Disorders

*Tracy Ssali^1,2^, Udunna C Anazodo^1,2^, Lucas Narciso^1,2^, Linshan Liu^1,2^, Sarah Jesso^1,3^, Lauryn Richardson^1,3^, Matthias Günther^4,5^, Simon Konstandin^4,6^, Klaus Eickel^6^, Frank Prato^1,2^, Elizabeth Finger^1,2,7^, Keith St Lawrence^1,2^

1. Lawson Health Research Institute, London, Canada

2. Department of Medical Biophysics, Western University, London, Canada

3. St. Joseph’s Health Care, London, Canada

4. Fraunhofer Institute for Medical Image Computing MEVIS, Bremen, Germany,

5. University Bremen, Bremen, Germany

6. Mediri GmbH, Heidelberg, Germany

7. Department of Clinical Neurological Sciences, Western University, London, Canada

# Supplementary Figures


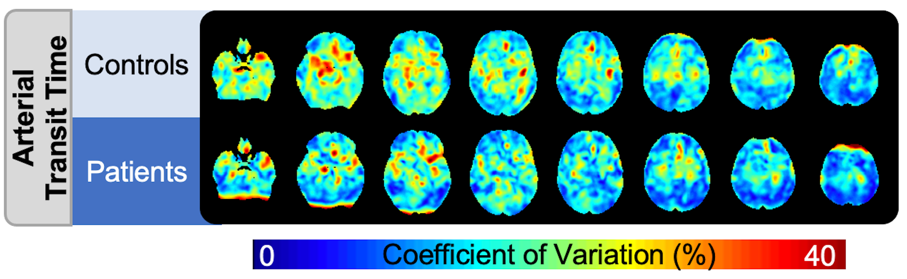


S Figure 1: Regional between-session reproducibility (coefficient of variation) of arterial transit times in controls and patients.
